# Editorial: Repurposed drugs targeting cancer signaling pathways: clinical insights to improve oncologic therapies, volume II

**DOI:** 10.3389/fonc.2025.1628842

**Published:** 2025-05-28

**Authors:** Alma D. Campos-Parra, Gerardo Leyva-Gomez, Teresita Padilla-Benavides

**Affiliations:** ^1^ Instituto de Salud Pública, Universidad Veracruzana, Xalapa, Mexico; ^2^ Departamento de Farmacia, Facultad de Química, Universidad Nacional Autónoma de México, Ciudad de Mexico, Mexico; ^3^ Molecular Biology and Biochemistry Department, Wesleyan University, Middletown, CT, United States

**Keywords:** drug repositioning, cancer, signaling pathways, treatments, personalize therapy

## Introduction

Drug repurposing in oncology is a strategy that attempts to identify new therapeutic uses of drugs already approved for other diseases to treat cancer. This strategy has gained interest because of its potential to reduce costs and accelerate the development of oncology treatments. This Research Topic aims to provide information on repositioned drugs in different types of cancer, to personalize and improve cancer therapies. Ten manuscripts in this Research Topic examine various but interconnected aspects of drug repurposing, highlighting the rapid advancement of the field and increasing complexity.

## Repositioning existing drugs for the treatment of common cancers

Common tumors refer to cancers with high incidence and prevalence across global populations, making them some of the most frequently diagnosed malignancies. These typically include solid tumors such as breast, lung, colorectal, prostate, ovarian, pancreatic, liver, and bladder cancers. Characterized by well-established clinical and biological profiles, these tumors are often supported by robust preclinical models and extensive clinical trial data. Due to their prevalence and clinical impact, they are strong candidates for drug repurposing to enhance outcomes, particularly in resistant cases or when treatment options are limited.

In this sense, triple negative breast cancer (TNBC) is usually an aggressive and difficult-to-treat cancer. On this topic, Carrion-Estrada et al. demonstrated a compelling strategy for targeting TNBC by stabilizing the oncogenic K-Ras4B G13D/PDE6δ complex using novel compounds (C14 and P8). These agents suppressed tumor growth in both *in vitro* and *in vivo* models, including resistant TNBC subtypes, highlighting their potential as adjuvant treatments when standard therapies fail. In a related effort to expand treatment options through drug repositioning, Hajihosseini et al. conducted a meta-analysis showing that olaparib, typically used in BRCA1/2-mutant breast and ovarian cancer, improved progression-free survival when used as monotherapy in lung cancer compared to combination regimens with durvalumab or gefitinib. In parallel, Pernot et al. explored an immunomodulatory approach through the repurposing of sulconazole, an antifungal compound that inhibits PD-1 expression in immune and cancer cells by blocking NF-κB and calcium signaling. The ability of sulconazole to restore immune activity while repressing malignant traits further highlights the value of nontraditional compounds in oncology, especially for immunologically evasive tumors (https://doi.org/10.3389/fimmu.2023.1278630).

Complementing these findings, Villegas-Vázquez et al. provided a comprehensive review on drug repositioning for ovarian cancer, emphasizing the critical role of cell line and animal models in preclinical drug screening. Although clinical application remains in early stages, these models are key to developing future therapies aimed at improving outcomes in patients with gynecologic cancers.

At the genomic level, Martinez-Montiel et al. discussed a paradigm shift by focusing on alternative splicing events in prostate cancer. As splicing errors increasingly emerge as hallmarks of malignancy, this review advocates for the development of diagnostics and therapies that target cancer-specific splicing isoforms, an especially timely strategy given the rising global burden of disease in low-resource settings. Additionally, Sánchez-Marín et al. discussed thyroid cancer at the genomic levels and identified 13 genes with missense mutations and 10 for gene fusions as potential therapeutic targets for drug repositioning. This which represents promising area for therapeutics, as treatment for this cancer is limited.

## Therapeutic opportunities through drug repositioning in uncommon cancers

Uncommon tumors are rare cancers with limited epidemiological data, including sarcomas, neuroendocrine tumors, certain pediatric cancers, and site-specific malignancies. Their low incidence often leads to underrepresentation in clinical trials and reliance on limited or extrapolated treatment evidence. Their rarity poses challenges for diagnosis, research, and treatment development, but also makes them ideal candidates for drug repurposing, offering quicker, cost-effective options where standard therapies are limited or ineffective.

Osteosarcoma is a rare and aggressive bone cancer, with complex diagnosis and treatment due to tumor heterogeneity. Despite its prevalence across age groups, comparative genomic data has been limited. A study by Zou et al. analyzed 194 patients and found age-related molecular differences. While common mutations like TP53 appeared across all ages, younger patients had more gene amplifications and homologous recombination deficiency, whereas adults had higher tumor mutational burden. Children showed more angiogenesis-related mutations, while older groups had alterations in PI3K/mTOR and cell cycle pathways. Notably, 58% of patients had actionable mutations, with treatment targets varying by age.

Giant cell tumor of bone (GCT) is a rare neoplasm with limited treatment options. In this context, McAllister et al. were the first to report the expression of Prostate-Specific Membrane Antigen (PSMA) in GCT. PSMA is the molecular target for the radioligand therapies Locametz and Pluvicto, currently approved for prostate cancer. Based on their findings, the authors suggest the potential repositioning of Locametz and Pluvicto as therapeutic options for GCT.

A broader overview of glioblastoma therapy shed light on the systemic challenges in treating this notoriously intractable disease. The review by Han Bae et al. emphasize the importance of biomarker discovery and innovative drug delivery technologies (e.g., nanoparticles, focused ultrasound) alongside drug repurposing, a strategy echoed in the sulconazole and C14/P8 studies, for overcoming barriers like the blood-brain barrier and tumor heterogeneity.

Finally, home chemotherapy initiatives have emerged as a viable and safe alternative to traditional hospital treatment for oncology patients, a safe alternative that could reduce costs and hospital burden. In this regard, Villegas et al. presented several recommendations based on the published literature and an expert panel in order to have a basis for the development of future initiatives, as they represent a new model of patient-centered oncology care.

## Summary and concluding remarks

This growing body of research emphasizes the promise of drug repurposing as a faster, cost-effective way to develop new cancer therapies by using existing drugs with known safety profiles. It highlights a shift toward targeted, mechanism-based treatments, aiming to improve outcomes, especially for difficult or understudied cancers. By combining molecular insights with innovative therapeutic approaches, these studies move the field closer to personalized and effective cancer care.

